# Removal of a Portion of a Catheter from the Bladder

**Published:** 1878-08

**Authors:** 


Removal of a Portion of a Catheter from the Bladder.—A man was admitted stating that he had suffered from atony of the bladder, and in his attempts to pass a catheter had left half of the instrument in the bladder. The accident happened six days before he presented himself, and had not caused any urgent symptoms. An examination by the sound showed the presence of a foreign body. The bladder was then injected, and, on the fluid coming out, the portion of the catheter came out with it. It proved to be half of an elastic catheter, covered with phosphates, and corresponding to No. 7 of the French scale.—*N. Y. Med. Jour.*

